# How Should Athletes Coping With COVID-19: Focus on Severity and Psychological Support

**DOI:** 10.3389/fpsyg.2021.559125

**Published:** 2021-06-24

**Authors:** Qi Han, Xueyang Li, Zhenghanxiao Wang

**Affiliations:** ^1^Sport Science College, Beijing Sport University, Beijing, China; ^2^Department of Sports Nutrition Research Center, National Research Institute of Sports Medicine, Beijing, China; ^3^Department of Psychology, University of Limerick, Limerick, Ireland; ^4^School of Psychology, Beijing Sport University, Beijing, China; ^5^Department of Academic Affairs, Yanqi Lake Beijing Institute of Mathematical Sciences and Applications, Beijing, China

**Keywords:** athletes, COVID-19, severity, mortality, psychology

## Abstract

**Objective:** Athletes are suffering from many uncertainties and hope to achieve the best possible position under the current circumstances of this global coronavirus disease (COVID-19) pandemic. In this study, we aimed to address the severity and psychological support for athletes with COVID-19.

**Methods:** We extracted public data and news reports of the up-to-date first seven cases of elite athletes with COVID-19 confirmed in China and made psychological recommendations based on scientific evidence.

**Results:** The severity and mortality in athletes who tested positive to COVID-19 are mild and extremely low. The included cases from different sports are two soccer players, two athletes from ice hockey, and three from fencing. In this study, we adapted well-recognized psychological questionnaires, improvised it for athletes to use under the COVID-19 pandemic, and also provided recommended psychological support.

**Conclusion:** The severity and mortality in Chinese athletes contracted with COVID-19 are mild and low with zero death. Psychological support of any kind from nurses, team medical staff, psychologists, family, and friends through social media and telecommunication should be adopted and can be of great help.

## Introduction

As was reported about a novel coronavirus disease (COVID-19) outbreak first in China (Zhu et al., 2020) and now across the nations and continents, we are currently witnessing a global challenge against the pandemic, the COVID-19 outbreak, all around the world (Spinazze et al., 2020; Spiteri et al., 2020). WHO announced the COVID-19 as a global public health emergency on January 30, 2020 and declared the “pandemic state” (Cucinotta and Vanelli, 2020; Mahase, 2020) on March 11, 2020. The organizing committee of Olympic Games Tokyo 2020 has gone through a series of discussions with the International Olympic Committee (IOC) and postponed the event for the first time in their history (Gallego et al., 2020). The opening ceremony of the Olympic Games Tokyo 2020 will now be rescheduled to July 23, 2021. Elite athletes who are readily prepared for participating in the Olympic Games 2020 are fully aware of the significance of its uncertainty due to the detraining effects on performance. Every athlete participating in Olympic Games hopes to achieve the best possible position for the once-in-a-four-year competition under the current circumstances of the global pandemic.

Professional athletes are usually young and physically active populations, and the symptoms, morbidity, and mortality observed in the elderly were almost trivial or not even existing in athletes. However, several specific issues raised during this pandemic situation hit the communities of athletes globally. From the perspective of decreasing the potential transmission of the coronavirus, lots of sports events were either postponed or canceled. For instance, though lots of teams requested athletes to stay home and keep physical fitness by training themselves or resume routine training in the facilities, lots of competitions are stopped or are played behind closed doors.

To our knowledge, currently, there are no specific data available regarding the prevalence, severity, and recommendations of psychological support for professional athletic individuals with COVID-19 (Hull et al., 2020). In this study, we aim to illustrate the severity of seven professional athletes with COVID-19 reported in China from March 12, 2020, to April 18, 2020 and demonstrate the recommended psychological support for athletes coping with COVID-19.

## Materials and Methods

### Data Sources of Professional Athletic Individuals Who Tested Positive

In the qualitative analysis of this study, seven professional elite athletes were officially reported by news media with confirmed positive COVID-19 tests taken in China from March 12, 2020, to April 18, 2020. Specimens of respiratory tests were collected by disease control or medical staff from local coronavirus testing facilities and double confirmed by the Centers for Disease Control and Prevention (CDC). The epidemiological data that officially released are described in Table 1.

**Table 1:** Characteristics of seven confirmed professional elite athlete patients with COVID-19.

Team	Age	N	Male	Female	S	Severity	Mortality	T
Soccer-CSL	32	1	1	0	NA	Mild	None	NA
Soccer-A series	30	1	1	0	42	Mild	None	NA
Epee fencing	NA	3	NA	NA	13	Mild	None	NA
Ice hockey	NA	2	0	2	11	Mild	None	NA

NA, not applicable; N, number of confirmed cases; T, temperature in Celsius; and S, stayed with a team of how many people.

Diagnosis, clinical classification criteria, and treatment plan and priority were based on the Diagnosis and Clinical Classification Criteria and Treatment Protocol of Novel Coronavirus Pneumonia (COVID-19) released by the National Health Committee of the People’s Republic of China (trial version VII, March 3, 2020; Wang et al., 2020). A confirmed case is based on epidemiological history (including cluster transmission), clinical manifestations (fever and respiratory symptoms), lung imaging, and PCR results of detection of coronavirus nucleic acid and testing of serum-specific antibodies. The clinical classification of severity in trial version VII is as follows: Mild cases are people having mild clinical symptoms, and no pneumonia manifestations can be found in imaging. Moderate cases have symptoms such as fever and respiratory tract symptoms, and pneumonia manifestations can be seen in imaging. Severe cases are those who meet any of the following criteria: respiratory rate ≥ 30 breaths/min; oxygen saturation ≤ 93% at a resting state; arterial partial pressure of oxygen (PaO_2_)/oxygen concentration (FiO_2_) ≤ 300 mmHg [while for high altitude district over 1 km, PaO_2_/FiO_2_ multiplies (local air pressure in mmHg/760 mmHg), where 1 mmHg = 0.133 kPa]. Patients with >50% progression of lesions within 24–48 h in lung imaging should be treated as severe cases. Critical cases are those that had any of the following symptoms: occurrence of respiratory failure that requires mechanical ventilation; the presence of shock; and failure(s) of other organs that requires monitoring and treatment in the intensive care unit (ICU).

Psychological support and recommendations are made and designed by psychiatry counseling personnel in accordance with the position stand of the American Medical Society for Sports Medicine (AMSSM, 2019) Position Statement (Chang et al., 2020) on Mental Health Issues and Psychological Factors in Athletes.

### Statistical Analysis

Results are expressed as mean ± SD, median [interquartile range (IQR)], or percentage where appropriate. We do not aim to look at the differences between groups; therefore, neither ANOVA nor Student’s *t*-tests were performed.

## Results

### Cases From Public Data

From public data posted on news media, seven professional elite athletes who have been diagnosed with COVID-19 in China were included in this study. Their characteristics are listed in Table 1.

### Mental Health Support

As it was reported, suspending sports competitions, detraining, receiving part of salaries, and even temporary layoff happened occasionally around the world, which put athletes into a stressful state across borders and nations. Monitoring psychological states and mental health of athletes and providing psychological intervention accordingly are of great importance in this situation. Quarantined and isolated from team, family, and friends also have psychological impacts on athletes, which were recognized to be associated with feelings of loneliness, anxiety, frustration, depression, etc. We acknowledge that Self-Reporting Questionnaire 20 (SRQ-20), Patient Health Questionnaire 9 (PHQ-9), Profile of Mood States (POMS; Rossi and Pourtois, 2012), Generalized Anxiety Disorder 7 (GAD-7), Hamilton Depression Rating Scale (HAMD), and Hamilton Anxiety Rating Scale (HAM-A) are very well-documented questionnaires and tools accessing mental health of patients; herewith, we designed a brief and easy-to-use mental health risk factor measuring questionnaire based on psychological symptoms relevant to athletes in this specific outbreak of COVID-19, as shown in Table 2, to perform fast Mental Health Survey (MHS) for nurses, psychologists, therapists, and team medical staff, where score 0 stands for the most severe symptoms that have been noticed and score 5 stands for no such symptoms.

**Table 2:** Brief mental health risk factors quantifying table for athletes with COVID-19.

Score	Mood	Stress	Anxiety	Sleep disorder	Depression	Eating disorder	ADHD	Overtraining status	Detraining status
0									
1									1
2									
3									
4									
5	5	5	5	5	5	5	5	5	

Score 0 stands for the most severe symptoms that have been noticed, and score 5 stands for no such symptoms. ADHD, attention–deficit/hyperactivity deficit. For the cases included in this study, we rated their mental health risk factors based on their reports to the news media and they scored 41 (*n* = 5) on average. Two elite athletes from the Chinese Ice Hockey Association and three elite athletes from the Chinese Fencing Association officially reported mild symptoms with good physical and psychological status. No stress, anxiety, sleep disorder, depression, eating disorder, or ADHD were reported. However, during their quarantine at the hospital before returning to the regular team training, detraining was acceptable and no overtraining was reported.

The outbreak of COVID-19 became the most life-threatening health and safety emergency affecting athletes since World War II, postponing the Olympic Games Tokyo 2020 to the Summer of 2021 and potentially canceling if the pandemic of COVID-19 persists in the year of 2021 according to the chief of Tokyo 2020 Olympic Games Committee. In this study, we introduced the issues that may bother Chinese athletes with COVID-19 and provided psychological interventions that should be of help under the circumstances of mental consequences of quarantine and detraining, such as psychological fatigue, depression, anxiety from unprecedented public health pandemic, losing social and financial support, and unknown situations and challenges for the future. Therefore, adapting the psychological check-ins in addition to the regular physical examinations is of great value to monitoring the mental health of elite athletes contracting COVID-19 and then making timely psychological interventions accordingly. The Human Resources Development Center of the General Administration of Sport of China released Psychological Guidance on Competitive Athletes at the beginning of the COVID-19 outbreak, which provided methods for athletes to perform mindfulness training under mental fatigue, pressure, and anxiety. Furthermore, Beijing Sport University released 90 episodes of a special lecture on Psychological Adjustment of Athletes Preparing for Olympic Games facing COVID-19 through social media, helping athletes to cope with psychological issues under stress and depression.

In addition to the physiological issues and ambient environmental situations (e.g., temperature, humidity, noise, and change of climate), psychological factors may cause insomnia among athletes who contracted COVID-19 and their teammates due to overthinking of postponed/canceled competitions, may increase the uncertainty of upcoming budget and financial support, and may cause lack of confidence due to the consequence of detraining-related effects on exercise performance, anxiety, and pressure. From the psychological perspective, athletes with a sleeping disorder, anxiety, depression, and an eating disorder under this circumstance can be aided by performing efficient MHSs and mindfulness-based training, recommended as follows, that partially adapted from scientific evidence and recommendations (Jukic et al., 2020).

Motivate athletes to stay positive and acknowledge that this is so far the biggest public health challenge since World War II for all communities globally (Cosic et al., 2020).

Improve mental fatigue and anxiety status by professional psychologists through telecommunication (Li et al., 2019).

Educate athletes on how to get over the COVID-19 pandemic (e.g., adopting the importance of nutritional support to fight infection, personalized physical fitness, and recovery functional training) and how to protect themselves with preventive and hygiene behaviors (Midgley, 2003).

Provide social support to help athletes relieve pressures by their organization, team head coach, fitness coach, nutritionist, and medical staff (Mann et al., 2020).

Encourage athletes to reach out to their best friends when seeking mental support and help them feel less isolated, since family members, trustworthy friends, and mentors are good places for people to turn to (Bogels and Emerson, 2019).

Teach athletes skills for relieving pressure (Aspy and Proeve, 2017), such as reminding themselves the scenes of winning and success, progressive relaxation [including deep breathing, progressive muscle relaxation (PMR), and yoga], and positive reinforcement/affirmations.

## Discussion

Coronavirus disease has placed big concerns on sports communities due to lots of uncertainties. Besides professional elite athletes with COVID-19, athletes who contracted COVID-19 but who did not meet the inclusion criteria were excluded (e.g., teenage athletes, not illustrating the same protocol in clinical confirmation). To our knowledge, this is the first epidemiological research study identifying patients with COVID-19 with a competitive sports occupation of China. It is suggested that athletes do not have natural immunity from COVID-19, and it seems that male athletes do not have a higher chance of contracting this virus. Among the reported seven elite athletes, two male athletes from soccer and two female athletes from ice hockey reported, but three epee fencing athletes have to be updated. The number of teammates and team staff that six athletes had stayed with was 66 in total, while one soccer player did not join the team since his last international traveling.

Regular training does not suppress the immune system; however, during exhausting workload and training volume, the immune function can be transiently suppressed (Campbell and Turner, 2018). Athletes are more likely to progress upper respiratory tract infection (URTI) immediately after very intensive competition or training with the scientific evidence that some athletes are more prone to RTIs in vigorous sport (Midgley, 2003). From the epidemiological data of nonathletes, COVID-19 may associate with potential damages on cardiomyocytes and lungs, causing cytokine storm syndrome (CSS; Chau et al., 2020; Shi et al., 2020; Ye et al., 2020). To date, there are no data available for evaluating whether COVID-19 plays a detrimental role in the performance of athletes; since many elite athletes compete in national and world level competitive sports, their returning to play should be well evaluated and monitored in consideration of cardiac and pulmonary rehabilitation.

Finally, and most importantly, we acknowledge that, while this coronavirus caused many deaths worldwide, it seems that COVID-19 plays a mild detrimental effect on active athletes, and no deaths among active elite athletes contracting COVID-19 have been reported since its outbreak. However, contact sports often require athletes in close contact with teammates due to daily training and traveling together, which puts them at a high risk of contracting COVID-19 through transmission during routine physical activities. For instance, a football player belonging to Italian Serie A tested positive for COVID-19, and the following day, his five teammates and a team doctor tested positive for COVID-19 (Corsini et al., 2020). To ensure the mental health and comprehensive wellbeing of elite athletes, conducting MHS and providing psychological support constantly can be of great value. There are several specific issues that can potentially induce psychological stress surrounding athletes since the pandemic outbreak. For example, though many athletes are optimistic about this virus and even ask their organizations to cut their salary, losing the opportunity to participate in important events (e.g., cancellation/postponement, aging, and not being able to keep fit), losing their significant ones, unemployment, and budget cut when they were immediately stopped from routine training and competitions can all contribute to a bad mood. After the outbreak of COVID-19, some Chinese sports teams asked athletes to stay with the team and keep an outstanding strength and conditioning training wherever they are located. Meanwhile, lots of competitions were canceled or postponed and up to 14 days of quarantine from travel affecting their routine training and leaving the contracted and uncontracted athletes with excessive self-occupied time. In this study, we adopted AMSSM position stand (Chang et al., 2020) and developed a fast and easy-to-use mental health risk factor quantifying table for athletes coping with COVID-19, hopefully providing some clues for the clinical overall wellbeing and psychological status monitoring. Constant, timely, and easy-to-perform psychological support can help these people feel less lonely or isolated and make it easier for them to get through this.

Besides the included cases, the preparedness planning for athletes with COVID-19 should include Paralympic athletes with disabilities, and the organizations should support and ensure their needs are taken into consideration. As it was well recognized, virtual communication can help people feel less lonely and less isolated; therefore, remotely connecting them with family, friends, and psychological counseling through telephone, video chat, and other social media can play a big role in psychological support.

## Limitations

The biggest, and the first, limitation of this study is not being able to evaluate the current psychological status and the overall physical fitness of the included athletes due to the lack of data being reported. The second limitation is not being able to have the number of total athletes and their staff out there; therefore, we cannot calculate the prevalence of COVID-19-confirmed cases among professional sport occupational communities.

## Conclusion

In conclusion, the severity and mortality in Chinese athlete patients with COVID-19 are trivial and low with zero death. Psychological support of any kind from coaches, nurses, team medical staff, psychologists, family, and friends can be of great help; therefore, it is important to motivate and encourage athletes to stay optimistic in these circumstances.

## Data Availability Statement

Publicly available datasets were analyzed in this study. These can be found at:

Ice hockey (http://icehockey.sport.org.cn/announcement/2020/0329/313323.html; https://www.chinadaily.com.cn/a/202003/30/WS5e81506ca310128217282e9a.html).
Fencing (http://fencing.sport.org.cn/tzgg/2020/0320/312863.html; https://www.chinadaily.com.cn/a/202003/20/WS5e7419c3a310128217280a23.html).
Soccer CSL (http://sports.sina.com.cn/china/j/2020-04-16/doc-iircuyvh8146734.shtml; https://www.chinadaily.com.cn/a/202003/22/WS5e772b75a31012821728105c.html).
Soccer-A series (https://sports.163.com/20/0319/09/F82SAI2700058780.html; https://www.marca.com/futbol/liga-china/2020/03/18/5e72158d22601df30e8b45fb.html).

## Author Contributions

QH collected the public clinical and epidemiological data. ZW and XL processed the data and drafted the manuscript. All authors contributed to the article and approved the submitted version.

### Conflict of Interest

The authors declare that the research was conducted in the absence of any commercial or financial relationships that could be construed as a potential conflict of interest.
